# Positive Molecular Detection of *Rickettsia helvetica* in Great Tits From Central Poland

**DOI:** 10.1002/ece3.71535

**Published:** 2025-06-09

**Authors:** Jarosław Wawrzyniak, Joanna Strzelczyk, Cecilia Panek, Michał Glądalski, Adam Kaliński, Marcin Markowski, Joanna Skwarska, Piotr Zieliński, Jerzy Bańbura

**Affiliations:** ^1^ Faculty of Biology and Environmental Protection, Department of Experimental Zoology and Evolutionary Biology University of Lodz Lodz Poland; ^2^ Laboratory of Personalized Medicine and Laboratory of Biotechnology “Bionanopark” Ltd Lodz Poland; ^3^ Faculty of Biology and Environmental Protection, Department of Ecology and Vertebrate Zoology University of Lodz Lodz Poland

**Keywords:** *Parus major*, *Rickettsia*, ticks, urban ecology

## Abstract

Ticks spread to new habitats via wild mammals and birds, with urban green spaces potentially colonized through bird transportation. *Rickettsia* is a genus of bacteria that can cause diseases in humans and animals, which is often transmitted by ticks. This study investigated the presence of *Rickettsia* in the great tit ( *Parus major* ), a widespread Eurasian passerine bird, and in ticks attached to them. Samples were collected in three locations around Lodz, Poland: a suburban forest, an urban park, and green patches near the city center. Using Nested PCR (polymerase chain reaction), 73 samples of blood from birds and five ticks taken from great tits (attached to them) were tested for the presence of *Rickettsia* DNA. Six birds (8.2%) tested positive for *Rickettsia* spp., with detections across all locations. Sequencing confirmed the presence of *Rickettsia helvetica* , a known zoonotic species. None of the ticks tested positive for *Rickettsia*. These findings indicate that synanthropic bird species, like the great tit, may play a role in spreading *Rickettsia* bacteria into urban areas. This study highlights the potential importance of birds in the ecology of tick‐borne diseases in urbanized environments.

## Graphical abstract

Great Tits were trapped in central Poland from three different habitats: a suburban forest, an urban park, and green patches near the city center. *Ixodes ricinus* species and samples of birds blood were collected from birds. Blood samples and ticks were molecularly tested for the presence of Rickettsia. Tick infestation was low at 5.5%. Prevalence of Rickettsia spp. was 8% in Great Tits' blood, and there were no differences between study places. None of the ticks tested positive for Rickettsia. Sequencing indicated the presence of *Rickettsia helvetica* .

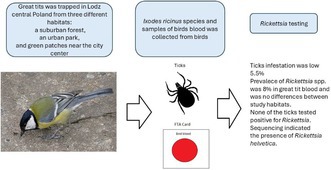

## 1 Introduction

Birds, especially passerines, often carry ticks during migration and breeding, facilitating the spread of tick and tick‐borne diseases (Hornok et al. 2013; Millins et al. 2018). Research has primarily focused on long‐distance migrants as tick transporters, and the prevalence of tick infestation depended mainly on the bird species and the foraging behavior (Olsén et al. 1995; Bjöersdorff et al. 2001; Hasle et al. 2009; Elfving et al. 2010). Also, local breeding populations of birds may be burdened with ticks to varying degrees depending on the habitat (Gregoire et al. 2002).

Ixodid ticks, which often occur on passerine birds, are important vectors and reservoirs for several human and animal pathogens, including *Rickettsia* spp. (Pfäffle et al. 2013). In Poland, *Ixodes ricinus* is the most common and widely distributed tick species, which is also found in urbanized areas and serves as the primary vector for several microbial pathogens (Welc‐Falęciak et al. 2014; Kowalec et al. 2019).

The genus *Rickettsia* includes species responsible for human diseases such as Rocky Mountain spotted fever and typhus. These bacteria are transmitted via arthropods, mainly ticks, which can serve as vectors and/or reservoirs for these organisms, spreading through transstadial and transovarial transmission (Perlman et al. 2006). However, the full range of *Rickettsia* reservoir hosts remains unclear, with ongoing research exploring the role of birds and other vertebrates (Hornok et al. 2013, 2014; Capligina et al. 2014; Lommano et al. 2014; Berthová et al. 2016). In natural vertebrate hosts, infections can result in rickettsiemia, allowing uninfected ticks to acquire the bacteria, thereby perpetuating the natural cycle. Reservoirs are organisms that can harbor and maintain the infectious agent over time, providing a source for its transmission to other hosts. Several studies have reported the presence of *Rickettsia* DNA in ticks collected from birds in various parts of Europe (Elfving et al. 2010; Socolovschi et al. 2012; Hornok et al. 2013; Capligina et al. 2014; Lommano et al. 2014; Mărcuţan et al. 2016) but relatively few have detected the presence of *Rickettsia* DNA in bird blood (Hornok et al. 2014; Berthová et al. 2016). Birds probably may act as incidental hosts for Rickettsia and may not be long‐term sources of infection for vectors due to a short period of bacteremia (Cardona‐Romero et al. 2022) Further research is needed to confirm whether birds can host for long time viable forms of Rickettsia and actively participate in its life cycle.

The great tit ( *Parus major* ) was selected as the target species for this study due to its widespread distribution and high abundance across Europe (EBCC 2022). As one of the most common and adaptable passerines, it inhabits a wide range of environments, including forests, suburban parks, and urban areas. Its ecological traits, such as frequent contact with vegetation and ground‐level foraging behavior, increase the likelihood of tick infestations, so it is often a host to immature stages of ticks (Klaus et al. 2016). These characteristics make it an appropriate model species for investigating the potential role of small passerines in the circulation of tick‐borne zoonotic pathogens. Rickettsia was chosen as the focal agent because it is a significant tick‐borne zoonotic pathogen reported in Europe, and there is limited information regarding its presence in avian hosts.

The main goal of this study was to evaluate the tick infestation in breeding populations of the great tit with respect to the prevalence of *Rickettsia*. We analyzed the occurrence of *Rickettsia* species in ticks attached to birds and in bird blood samples using molecular methods. This study was designed as a preliminary investigation to assess the occurrence of vector‐borne pathogens in a local population of *Parus major* during a single breeding season. The project was conducted within the framework of a small exploratory grant, and thus was limited in its temporal and logistical scope. Although preliminary in nature, the results provide an important foundation for future, broader‐scale research aiming to better understand host–vector–pathogen dynamics in wild bird populations.

## 2 Materials and Methods

### 2.1 Sample Collection

Adult great tits were captured throughout the breeding season, which spans from nest building to fledging, from April to early July of 2022, around the city of Lodz in central Poland at three locations: a rich deciduous forest (51°50′ N, 19°29′ E; study site 1), an urban parkland (51°76′ N, 19°41′ E; study site 2), and city green patches (51°46′N, 19°29′ E; study site 3). The forest study site is a 150 ha area in the interior of a mature mixed deciduous forest (1250 ha in total), adjacent to the northeastern suburbs of Lodz. Oak species, *Quercus robur* and *Quercus petraea* , predominate here. The urban parkland study site is located in the southwestern part of Lodz in the Botanical Garden, characterized by fragmented tree cover and a high proportion of non‐native plant species. The garden borders urbanized areas with apartment blocks to the south, southeast, and north. The city green patches study site is situated near the campus of the Faculty of Biology and Environmental Protection, the University of Lodz, and is surrounded by tall buildings, sidewalks, parking lots, streets with heavy traffic, and areas covered with trees and bushes. These three locations represent habitats with varying degrees of human transformation and anthropogenic pressure.

Birds were mist‐netted using mist‐nets to capture passerine birds (mesh size 14 × 14mm, height 2.5 m, long 6 m) within their breeding territories, attracted by playing male song through a loudspeaker. Playback was used in a limited and controlled manner to minimize disturbance, and birds were handled briefly and released immediately after sample collection to reduce stress. In a few instances near the end of the breeding season, adult birds were captured in nest boxes while feeding chicks around 2 weeks old. In the field, the sex of each great tit was determined, and all birds were checked for the presence of hard ticks (Acari: Ixodidae). Ticks were removed with fine forceps, placed in 70% ethanol in separate vials, and stored at room temperature. One immature tick could not be removed due to its location on the eyelid, near the eye, posing a high risk of injuring the bird. Ticks were morphologically identified to the genus and/or species level using identification keys (Nowak‐Chmura 2013). Unfortunately, the condition of the collected larva after removal from the bird did not allow for species‐specific identification. Species identification was based on morphological features, which remains an accepted and widely used method for tick identification. Nonetheless, we acknowledge that the use of complementary genetic methods would further enhance taxonomic resolution, and we recommend their inclusion in future research efforts, especially for immature stages of Ixodes spp. In the next step, the ticks collected from the birds were taken to the laboratory for appropriate preparation for molecular detection of Rickettsia DNA.

From each captured great tit (73 individuals), approximately 10–20 μL of blood was collected from the ulnar vein directly onto a QIAcard FTA Mini (Qiagen), which was dried and stored at room temperature for later analysis. Blood sampling was performed by a qualified ornithologist who holds certification and training in handling and collecting blood from wild birds, in accordance with Polish law regulations. The sampling procedure involved ulnar venipuncture using sterile techniques to minimize stress and risk to the birds. All procedures were conducted under ethical approval [permit numbers in section Ethical guidelines].

### 2.2 Laboratory Methods

Ticks and blood sample preparation DNA from ticks was isolated using the QIAamp DNA Blood and Tissue Kit protocol supplement Qiagen for purifying DNA from insects. The ticks were dried, washed with water and detergent, rinsed with tap water and distilled water, and then homogenized. After homogenization, 180 μL of ATL buffer and 20 μL of proteinase K were added to the samples, which were then incubated at 56°C. DNA was extracted from this lysate, and the DNA concentration was measured using a Qubit fluorometer (Life Technologies, Thermo Fisher Scientific).

DNA from blood samples from tits was isolated according to the QIAamp DNA Blood and Tissue Kit protocol (Qiagen). Fragments were cut from FTA cards under sterile conditions and lysed using 180 μL of ATL buffer and 20 μL of proteinase K. DNA was extracted from this lysate; DNA concentration was measured using a Nanovue spectrophotometer (VWR). The quality and integrity of the DNA were visualized on a 1% agarose gel using the Quick‐Load Purple 1 kb DNA Ladder (NEB), with band sizes ranging from 500 bp to 10 kb.

#### 2.2.1 PCR Analysis and Identification of *Rickettsia* Species

All DNA samples from bird blood and ticks were tested for the presence of *Rickettsia* using a nested PCR method targeting the 17 kDa *Rickettsia* antigen gene. The 17 kDa antigen gene, often referred to as ompA, encodes an outer membrane protein (OMP) of approximately 17 kDa. It is one of the major antigens of Rickettsia species and is expressed on the surface of the bacteria. This protein is involved in the adherence of the bacterium to host cells and plays a role in the pathogenesis of rickettsial infections. The 17 kDa protein is considered a highly conserved target for molecular identification, making it an excellent candidate for detection assays. The expression of 17 kDa is related to the rickettsial infection's ability to invade host cells, making it a reliable marker for detecting the pathogen in biological samples from hosts.

This protocol used the external primers published by (Webb et al. 1990): Primer1 (5′‐GCTCTTGCAACTTCTATGTT‐3′) and Primer2 (5′‐CATTGTTCGTCAGGTTGGCG‐3′), which generate a 434 bp fragment, along with the nested primers published by (Schriefer et al. 1994), Rick 1 (5′‐CATTACTTGGTTCTCAATTCGGT‐3′) and Rick 2 (5′ GTTTTATTAGTGGTTACGTAACC‐3′), which yield a 231 bp fragment. Both PCR reactions were carried out in 25 μL volumes using OneTaq Hot Start Quick‐Load 2X Master Mix with Standard Buffer (NEB) and 0.4 μM each of the forward and reverse primers with 100 ng genomic DNA in the primary PCR and 1 μL of PCR1 product in the nested PCR reaction. In the case of a positive result in the PCR1 reaction, the template was 10‐fold diluted. Negative controls (nuclease‐free water instead of DNA template) as well as a positive control (*Rickettsia* DNA obtained from the Department of Parasitology, Faculty of Biology, University of Warsaw) were included in all assays.

In the first PCR reaction, an initial denaturation at 94°C for 3 min was followed by 35 cycles with denaturation at 94°C for 30 s, annealing at 57°C for 45 s, and elongation at 68°C for 45 s, with a final elongation at 68°C for 7 min. The protocol for the nested PCR consisted of an initial denaturation at 94°C for 3 min, followed by 35 cycles with denaturation at 94°C for 30 s, annealing at 53°C for 30 s, and elongation at 68°C for 30 s, with final elongation at 68°C for 7 min. Both PCRs were performed on a SureCycler 8800 thermal cycler (Agilent Technologies).

Reaction products were visualized on a 2.5% agarose gel stained with ethidium bromide using the Quick‐Load Purple 50 bp DNA Ladder (NEB) as a marker. The products of positive samples were purified from the gel using the Monarch Gel Extraction Kit (NEB) according to the manufacturer's protocol and submitted for Sanger sequencing. The DNA sequences obtained were analyzed using the DNA Sequence Assembler (Heracle BioSoft S.R.L.) and searched against the GenBank database using Basic Local Alignment Search Tool (BLAST) analysis.

#### 2.2.2 Statistical Methods

Exact confidence intervals (CIs) for the prevalence rates at the 95% level were calculated according to the Clopper‐Pearson method using the free software Quantitative Parasitology 3.0 (Reiczigel et al. 2019). The sample prevalence data was analyzed using the chi‐square test in the case of prevalence among the three study sites. Differences were considered significant when *p* < 0.05.

## 3 Results

### 3.1 The Great Tit and Tick Infestation

A sample of 73 great tits was captured during the breeding season in three locations in the city of Lodz (Table 1). The prevalence of tick infestation was 5.5% [4/73] (Cl: 1.5%–13.4%). The number of ticks per infested bird ranged from 1 to 2; two individual great tits were infested with two ticks each, and two individuals were infested with one tick each (mean intensity 1.5). All ticks were located on the head, in areas around the eyes or on a side of the beak. A total of five ticks were removed from three tits. One tick (probably a larva) was not removed from one individual due to its location on the eyelid. Among the five sampled ticks, four were nymphs identified as *Ixodes ricinus* , while one larva was classified at the genus level as *Ixodes*.

**TABLE 1:** Number of Great Tits caught and screened for attached ticks at each study site, and prevalence of tick infestation.

	Male (tick infested/all)	Female (tick infested/all)	Total
Rich deciduous forest site	2/18	0/7	2/25
Urban parkland site	0/18	1/7	1/25
City Green Patches site	1/18	0/5	1/23
Total	3/54	1/19	4/73

### 3.2 Molecular Detection of Bacteria in Ticks Collected From Birds and in the Blood of Great Tits

All collected ticks (five specimens: four nymphs and one larva from three great tits individuals) were tested for the presence of *Rickettsia*, but none was positive. The larva and the nymph collected from a *Rickettsia*‐positive bird proved free of *Rickettsia* either. Among the 73 blood‐sampled great tits, six were found to be nested PCR‐positive with *Rickettsia* spp. (prevalence 8.2%; Cl: 3%–17%), four from the deciduous forest site (prevalence 16%; Cl: 4.5%–36.1%) and one each from the urban parkland site (prevalence 4%; Cl: 0.1%–20.4%) and the city green patches site (prevalence 4.3%; Cl: 0.1%–21.9%). The differences between study areas were not statistically significant (*χ* ^2^ = 3.05; df = 2, *p* = 0.217). All *Rickettsia*‐positive birds were captured in April. The DNA samples from the six *Rickettsia*‐positive blood samples analyzed for nucleotide sequences and compared to those available in the GenBank database. To obtain a single consensus sequence, the “forward” and “reverse” reads were assembled into a contig. The obtained sequences in FASTA format were then analyzed using nucleotide BLAST (blastn) on the NCBI website (https://blast.ncbi.nlm.nih.gov/). The search was performed against the database using “highly similarity sequences megablast” search parameters. Matches were considered as identified if they met the following criteria: Identity percentage ≥ 97%; Query coverage ≥ 95%. Species names were assigned based on the best match (highest % identity and coverage). Four samples showed 97%–99% similarity to *R. helvetica*, (Table 2). Two samples did not meet the established criteria, so was classified us undetermined *Rickettsia* spp.

**TABLE 2:** Closest BLAST matches, and identity percentages for the17 kDa antigen gene, from great tit blood samples K2Z6482, K2Z4207, K2Z6470, and K2Z6477. Sequences for Rickettsia spp. were identified and characterized, with the closest matches corresponding to *R. helvetica* .

Gene	Sample ring no	Forward sequence rick1	Reverse sequence rick2	Contig sequence	Closest match	Identity [%]
17 kDa antigen gene	K2Z6482	GCGATGGCACTTGTCGGAGTAGGTGTAGGTGCATTACTTGGAGCAGTTCTTGGCGGGCAAATCGGTGCAGGTATGGATGAGCAGGATAGAAGACTTGCAGAGCTTACCTCACAGAGAGCTTTAGAAGCAGCTCCTAGCGGTAGTAACGTARAGTGGCGTAATCCGGATAACGGCAATTACGGTTACGTAACCACTAATAAAAAAA	ACCCTTAATACGGCTTGTTTTCGTTCGATCGCMCTCTACGTTACTACCGCTAGGAGCTGCTTCTAAAGCTCTCTGTGAGGYAAGCTCTGCAAGTCTTCTATCCTGCTCATCCATACCTGCACCGATTTGCCCGCCAAGAACTGCTCCAAGTAATGCACCTACACCTACTCCGACAAGTTGCCCTTTACCTTTACCGAATTGAGAACCAAGTAATGA	TCATTACTTGGTTCTCAATTCGGTAAAGGCAAGGGCACTTGTCGGAGTAGGTGTAGGTGCATTACTTGGAGCAGTTCTTGGCGGGCAAATCGGTGCAGGTATGGATGAGCAGGATAGAAGACTTGCAGAGCTTACCTCACAGAGAGCTTTAGAAGCAGCTCCTAGCGGTAGTAACGTAGAGNGCGATCGGAAAACAGCAATTAAGGGTACGTAACCACTAATAAAAAAA	*Rickettsia helvetica*	98
17 kDa antigen gene	K2Z4207	GCCCTGGCACTTGTCGGAGTAGGTGTAGGTGCATTACTTGGAGCAGTTCTTGGCGGGCAAATCGGTGCAGGTATGGATGAGCAGGATAGAAGACTTGCAGAGCTTACCTCACAGAGAGCTTTAGAAGCAGCTCCTAGCGGTAGTAACGTARAGTGGCGTAATCCGGATAACGGCAATTACGGTTACGTAACCACTAATAAAAC	ACATAATACGGCTTGGTCGTTCGATCGCACTCTACGTTACTACCGCTAGGAGCTGCTTCTAAAGCTCTCTGTGAGGTAAGCTCTGCAAGTCTTCTATCCTGCTCATCCATACCTGCACCGATTTGCCCGCCAAGAACTGCTCCAAGTAATGCACCTACACCTACTCCGACAAGTTGCCCTTTACCTTTACCGAATTGAGAACCAAGTAATAA	TTATTACTTGGTTCTCAATTCGGTAAAGGCAAAGGCACTTGTCGGAGTAGGTGTAGGTGCATTACTTGGAGCAGTTCTTGGCGGGCAAATCGGTGCAGGTATGGATGAGCAGGATAGAAGACTTGCAGAGCTTACCTCACAGAGAGCTTTAGAAGCAGCTCCTAGCGGTAGTAACGTAGAGTGCGATCGAACGACAAGCCGGTTACGTAACCACTAATAAAAC	*Rickettsia helvetica*	99
17 kDa antigen gene	K2Z6470	GCCGATGGGCACTTGTCGGAGTAGGTGTAGGTGCATTACTTGGAGCAGTTCTTGGCGGGCAAATCGGTGCAGGTATGGATGAGCAGGATAGAAGACTTGCAGAGCTTACCTCACAGAGAGCTTTAGAAGCAGCTCCTAGCGGTAGTAACGTARAGTGGCGTAATCCGGATAACGGCAATTACGGTTACGTAACCACTAATAAAAAAA	ACCYTTATTACGGCTTGTCCGTTCGATMGCCACTCTACGTTACTACCGCTAGGAGCTGCTTCTAAAGCTCTCTGTGAGGTAAGCTCTGCAAGTCTTCTATCCTGCTCATCCATACCTGCACCGATTTGCCCGCCAAGAACTGCTCCAAGTAATGCACCTACACCTACTCCGACAAGTTGCCCTTTACCTTTACCGAATTGAGAACCAAGTAATGAA	TTCATTACTTGGTTCTCAATTCGGTAAAGCCAAAGGGCACTTGTCGGAGTAGGTGTAGGTGCATTACTTGGAGCAGTTCTTGGCGGGCAAATCGGTGCAGGTATGGATGAGCAGGATAGAAGACTTGCAGAGCTTACCTCACAGAGAGCTTTAGAAGCAGCTCCTAGCGGTAGTAACGTAGAGTGGCGATCCGGACAACCGCAATAACGGTTACGTAACCACTAATAAAAAAA	*Rickettsia helvetica*	97
17 kDa antigen gene	K2Z6477	CCCGGTTGGTCACATTGTCGGAGTAGGTGTAGGTGCATTACTTGGAGCAGTTCTTGGCGGGCAAATCGGTGCAGGTATGGATGAGCAGGATAGAAGACTTGCAGAGCTTACCTCACAGAGAGCTTTAGAAGCAGCTCCTAGCGGTAGTAACGTARAGTGGCGTAATCCGGATAACGGCAATTACGGTTACGTAACCACTAATAAAAC	ACCCTTATACGGCTCGTCGTCGGATCGCACTCWMGTTAMTACCGCTAGAGAGCTGCTTCTAAAGCTCTCTGTGAGGYAAGCTCTGCAAGTCTTCTATCCTGCTCATCCATACCTGCACCGATTTGCCCGCCAAGAACTGCTCCAAGTAATGCACCTACACCTACTCCGACAAGTTGCCCTTTACCTTTACCGAATTGARAACCAAGTAAKA	TTTACTTGGTTYTCAATTCGGTAAACCGAAAGGGCAATTGTCGGAGTAGGTGTAGGTGCATTACTTGGAGCAGTTCTTGGCGGGCAAATCGGTGCAGGTATGGATGAGCAGGATAGAAGACTTGCAGAGCTTACCTCACAGAGAGCTTTAGAAGCAGCTCCTAGCGGTAGTAACGNAAGTGCGATCCGACAACGGCAATTAAGGGTACGTAACCACTAATAAAAC	*Rickettsia helvetica*	99

## 4 Discussion

This study focused on the Great Tit during the breeding season in three sites with varying degrees of urbanization. Unlike most studies that focus on bird migration, we investigated tick infestation and the presence of *Rickettsia* spp. DNA in both ticks attached to the birds and in the birds' blood. Great tits, generally short‐distance migrants or sedentary, are known to inhabit diverse environments (Gosler 1993), providing a suitable model for examining tick‐borne pathogen dynamics.

Tick infestation rates were low, with only 5.5% of Great Tits hosting immature *Ixodes ricinus* ticks. Infested birds were found in all three habitats, with no significant differences in infestation rates or intensity across sites. This aligns with studies indicating that tick infestation in birds depends on various factors, including bird species, feeding behavior, habitat characteristics, host density, and environmental conditions such as microclimate (Gregoire et al. 2002; Pfäffle et al. 2013).

Studies across Europe have documented tick infestation patterns in birds during migration, with rates similar to ours: ~3% in Germany (Klaus et al. 2016) and southern France (Socolovschi et al. 2012), slightly above 7% in Norway (Hasle et al. 2009), and higher variability during breeding seasons. For instance, a Polish forest study reported over 43% of birds infested, dominated by *I. ricinus* , and primarily affecting ground‐foraging species (Biernat et al. 2016). Tick infestation in Great Tits is variable. For instance, this species was the most infested bird species among 16 others, with almost 84% of individuals infested across three study areas in Slovakia (Berthová et al. 2016). Similarly, in Germany (Klaus et al. 2016) identified the Great Tit as one of the most heavily infested bird species. Conversely, urban studies, such as those in Bratislava, Slovakia, showed a much higher tick load on Great Tits than observed in our study (Chvostáč et al. 2018). However, in some studies, the Great Tit is not listed among the bird species frequently infested by ticks (Hasle et al. 2009; Socolovschi et al. 2012). Seasonal and local factors strongly influence tick infestation (Pfäffle et al. 2013); also, our previous occasional observations suggested the possibility of large year‐to‐year fluctuations, highlighting the need for long‐term studies to clarify these dynamics.

Molecular evidence of *Rickettsia helvetica* , a spotted fever pathogen associated with human infections, was detected in the blood of Great Tits in this study. Similar findings via PCR have been reported in Europe, with *Rickettsia* spp. prevalence in bird blood samples ranging from 1.5% to 8.9% (Ioannou et al. 2009; Hornok et al. 2014; Berthová et al. 2016). Notably, all birds with *Rickettsia* DNA in our study were captured in April, consistent with findings from Hungary and Slovakia, where *R. helvetica* was the dominant strain. In Slovakia (Berthová et al. 2016) reported a similar prevalence of *Rickettsia* spp. DNA in bird blood samples (8.9%), with *R. helvetica* being the most frequently identified species. The Great Tit was a bird species that had the most frequent positive results among the nine (*P.major, Sylvia atricapilla, Cyanistes caeruleus, Fringilla coelebs, Prunella modularis, Turdus merula, Erithacus rubecula, Emberiza schoeniclus, Passer montanus*) that tested positive for *Rickettsiae*, among 336 blood samples from 43 bird species. Additionally, it was also the most heavily infested with *I. ricinus* , which carried *R. helvetica* (Berthová et al. 2016).

However, no *Rickettsia* spp. DNA was detected in the ticks attached to birds in our study, contrasting with other research showing *Rickettsia* infection rates in ticks ranging from 6.6% to over 51% (Hornok et al. 2014; Berthová et al. 2016). Previous studies suggest that *Rickettsia*‐positive birds may infect ticks with low efficiency, but their role in the pathogen's life cycle remains unclear. Birds might act as carriers, spreading infected ticks to new locations, particularly in urban environments, but to confirm whether *Rickettsia* DNA‐positive birds are directly linked to the *Rickettsia* spp. cycle in nature, more specific research is needed.

Throughout Europe, various *Rickettsia* species have been identified in ticks attached to birds (e.g., Elfving et al. 2010; Socolovschi et al. 2012; Klaus et al. 2016; Biernat et al. 2016; Chvostáč et al. 2018). Despite the widespread distribution of these pathogens, research on *Rickettsia* spp. in bird blood remains scarce, with limited understanding of their pathogenicity to birds or their role in the pathogen's transmission cycle. Synanthropic birds, such as great tits, may facilitate the spread of infected ticks across habitats, but more specific research is needed to confirm their ecological role as host for Rickettsia. Although birds can act as hosts for certain Rickettsia species and may contribute to the life cycle of some Rickettsia species by serving as hosts for arthropods that transmit the bacteria, they can only remain rickettsemic for short periods (Parola et al. 2013). Detecting pathogen DNA in bird blood samples does not necessarily imply that the species plays an active role as a reservoir or amplifier in the pathogen's epidemiological cycle. We emphasize that DNA detection may reflect only transient infection, mechanical carriage, or spillover exposure from vectors, without true competence for maintaining or transmitting the pathogen. Confirmation of a species' role in pathogen transmission would require further evidence, such as isolation of viable bacteria, demonstration of pathogen replication in the host, or experimental transmission studies.

This study highlights the low prevalence of tick infestation and *Rickettsia* DNA in great tits during the breeding season and the variability of these factors across habitats. While *R. helvetica* was detected in bird blood, its absence in attached ticks suggests complex interactions between birds, ticks, and pathogens. One limitation of this study is the relatively small sample size and the restriction to a single breeding season. Additionally, the low number of ticks collected from birds was unexpectedly lower compared to our previous field observations, where higher levels of tick infestation had been recorded. This variability is consistent with reports from the literature, which highlight significant seasonal and interannual fluctuations in ectoparasite prevalence (Pfäffle et al. 2013). These findings underscore the importance of conducting multi‐seasonal and long‐term studies to obtain more comprehensive insights into infestation rates and pathogen circulation dynamics. Another limitation of our study is the lack of genetic identification for the larval tick specimen. Given the morphological similarities between immature stages of Ixodes species, future research should combine morphological and molecular approaches to improve taxonomic resolution and enhance the accuracy of vector‐pathogen association studies.

### 4.1 Ethical Guidelines

Bird catching and handling was conducted according to Polish legislation in this area. Following the collection of blood samples, all captured birds were safely released. The sampling techniques and all procedures were approved by the Local Bioethical Commission for Experiments on Animals, Medical University in Lodz (No. 70/ŁB07/2015 and No. 55/ŁB07‐UZ‐PTP/2021) and the Regional Directorate for Environmental Protection (WPN.6401.21.2022.BWo.).

## Author Contributions

**Jarosław Wawrzyniak:** conceptualization (lead), data curation (equal), formal analysis (equal), funding acquisition (lead), investigation (equal), methodology (supporting), project administration (lead), resources (equal), software (equal), supervision (equal), validation (equal), writing – original draft (lead), writing – review and editing (equal). **Joanna Strzelczyk:** conceptualization (supporting), investigation (equal), methodology (lead), project administration (supporting), resources (equal), writing – original draft (supporting). **Cecilia Panek:** conceptualization (supporting), investigation (equal), methodology (lead), project administration (supporting), resources (equal). **Michał Glądalski:** conceptualization (supporting), data curation (equal), investigation (equal), project administration (supporting), resources (equal), supervision (equal), validation (equal), writing – review and editing (supporting). **Adam Kaliński:** conceptualization (supporting), data curation (equal), investigation (equal), project administration (supporting), resources (equal), supervision (equal), validation (equal), writing – review and editing (supporting). **Marcin Markowski:** conceptualization (supporting), data curation (equal), investigation (equal), project administration (supporting), resources (equal), supervision (equal), writing – review and editing (supporting). **Joanna Skwarska:** conceptualization (supporting), data curation (equal), investigation (equal), project administration (supporting), resources (equal), supervision (supporting). **Piotr Zieliński:** conceptualization (supporting), data curation (equal), resources (equal), writing – review and editing (supporting). **Jerzy Bańbura:** conceptualization (equal), data curation (equal), formal analysis (supporting), investigation (equal), project administration (supporting), resources (equal), supervision (equal), validation (equal), writing – review and editing (lead).

## Conflicts of Interest

The authors declare no conflicts of interest.

## Data Availability

The data that support the findings of this study are available in figshare reference URL: https://figshare.com/articles/dataset/J_W_Datasets_Rickettsia_E_E_xlsx/28333727?file=52100279. Data file available on online repository Figshare using link 10.6084/m9.figshare.28333727.
